# Limited Dissemination of Extended-Spectrum β-Lactamase– and Plasmid-Encoded AmpC–Producing *Escherichia coli* from Food and Farm Animals, Sweden

**DOI:** 10.3201/eid2204.151142

**Published:** 2016-04

**Authors:** Stefan Börjesson, Sofia Ny, Maria Egervärn, Jakob Bergström, Åsa Rosengren, Stina Englund, Sonja Löfmark, Sara Byfors

**Affiliations:** Author affiliations: National Veterinary Institute, Uppsala, Sweden (S. Börjesson, S. Englund);; Public Health Agency of Sweden,; Stockholm, Sweden (S. Ny, J. Bergström, S. Löfmark, S. Byfors);; National Food Agency, Uppsala (M. Egervärn, Å. Rosengren)

**Keywords:** Escherichia coli, bacteria, antimicrobial resistance, extended-spectrum β-lactamase, ESBL, plasmid-encoded AmpC, pAmpC, foodborne infections, farm animals, meat, broilers, poultry, bloodstream infections, carriage, extended-spectrum cephalosporin, national assessment, Sweden

## Abstract

Extended-spectrum β-lactamase (ESBL)– and plasmid-encoded ampC (pAmpC)–producing *Enterobacteriaceae* might spread from farm animals to humans through food. However, most studies have been limited in number of isolates tested and areas studied. We examined genetic relatedness of 716 isolates from 4,854 samples collected from humans, farm animals, and foods in Sweden to determine whether foods and farm animals might act as reservoirs and dissemination routes for ESBL/pAmpC-producing *Escherichia coli*. Results showed that clonal spread to humans appears unlikely. However, we found limited dissemination of genes encoding ESBL/pAmpC and plasmids carrying these genes from foods and farm animals to healthy humans and patients. Poultry and chicken meat might be a reservoir and dissemination route to humans. Although we found no evidence of clonal spread of ESBL/pAmpC-producing *E. coli* from farm animals or foods to humans, ESBL/pAmpC-producing *E. coli* with identical genes and plasmids were present in farm animals, foods, and humans.

## Abstract 2

Food is a limited source of these antimicrobial resistance genes for humans.

In 2012, the Panel on Biologic Hazards of the European Food Safety Authority (EFSA) concluded that a risk exists for transmission of extended-spectrum β-lactamase (ESBL)– and plasmid-encoded AmpC (pAmpC)–producing *Enterobacteriaceae* from farm animals, particularly poultry, to humans through the food chain (*1*). This conclusion is problematic because ESBL and pAmpC hydrolyze extended-spectrum cephalosporins, which are one of the most widely used antimicrobial drug classes (*2*). Extended-spectrum cephalosporins are also listed by the World Health Organization as being critically useful antimicrobial drugs in human medicine (*2*). Therefore, the high frequency of ESBL/pAmpC-producing *Escherichia coli* reported for farm animals, particularly broilers, in Europe is of great concern (*3*). A recent systematic review by Lazarus et al. (*4*) reported the same conclusion as the EFSA but also that the magnitude of transmission and its geographic extent are still unclear. In addition, these authors reported a lack of studies on a national level and no comparisons of isolates from animals or food with isolates from human asymptomatic carriage (*4*).

In Sweden, antimicrobial drugs are used less often in animals and humans than in other countries in Europe and the frequency of ESBL/pAmpC-producing *E. coli* is lower (*5**,**6*). However, one exception in Sweden is the large frequency of pAmpC-producing *E. coli* in poultry and domestic chicken meat (*7**–**10*). In addition, Sweden has a low population density of humans and animals. Therefore, the situation in Sweden with regards to dissemination of ESBL/pAmpC-producing *E. coli* could be different from that for previous studies from other countries in Europe, which reported potential transmission of ESBL/pAmpC-producing *E. coli* from farm animals by foods to humans (*11**–**13*).

The objective of this study was to investigate potential dissemination of ESBL/pAmpC-producing *E. coli* isolates among foods, farm animals, patients with bloodstream infections, and presumed healthy human carriers in the community in Sweden. This objective was achieved by comparing genetic relatedness of ESBL/pAmpC-producing isolates from foods intended for retail markets, farm animals, and humans in Sweden. The study was conducted on a national level, tested a large collection of isolates from diverse sectors, and was undertaken in cooperation with governmental agencies for human health, animal health, and food safety. We aimed to improve overall knowledge regarding the influence of farm animals and foods on the frequency of ESBLs and pAmpCs in humans.

## Materials and Methods

### Datasets and Isolates

**A total of 716 ESBL/pAmpC-producing *E. coli* isolates from 4,854 samples obtained during 2010–2013 were available for analysis (**Table 1**). Isolates were from community carriers (n = 103);** patients with **bloodstream infections (n = 387); broilers (n = 32), laying hens (n = 10), pigs (n = 3), calves (n = 9), and chicken meat (n = 74) from Sweden; imported chicken meat (n = 84); imported beef/pork (n = 16); and imported leafy greens (n = 2). ESBL/pAmpC-producing *E. coli* isolates have not been found in samples of beef or pork from Sweden** (*5*). **All isolates were obtained by using equivalent methods. Samples from farm animals and foods were screened by using cefotaxime, and samples from community carriers were screened by using cefpodoxime. Isolates from bloodstream infections have reduced susceptibility to ceftazidime or cefotaxime and were submitted to the Public Health Agency of Sweden by 18 clinical microbiology laboratories (S. Ny et al., unpub. data).**

**Table 1:** Characteristics of *Escherichia coli* isolates from various sources tested for ESBL and pAmpC, Sweden*

Category and source	Year	No. samples	% Positive for ESBL	% Positive for pAmpC	Reference
Human					
Community carriers	2011–2013	2,134	4.3	0.4	S. Ny et al., unpub. data
Bloodstream infections	2011–2012	387†	92.5	7.5	S. Ny et al., unpub. data
Farm animals					
Broilers	2010	100	6.0	28.0	(*7**,**9*)
Laying hens	2012	69	4.4	8.7	(*14*)
Pigs	2011	184	1.6	0	(*14*)
Calves	2011–2012	729	0.7	0.5	(*15*)
Meat					
Domestically produced chicken	2010	100	4.0	40.0	(*8*)
Domestically produced chicken	2013	59	0	50.8	(*14*)
Imported chicken (Europe)	2010–2011, 2013‡	109	19.3	21.1	(*10*), S. Börjesson et al., unpub. data
Imported chicken (South America)	2010–2011	43	90.7	4.7	(*10*)
Imported beef (Europe)	2010–2011	136	5.9	0	(*10*)
Imported beef (South America)	2010–2011	42	0	0	(*10*)
Domestically produced pork	2011	100	0	0	(*14*)
Imported pork (Europe)	2010–2011	119	5.9	0.8	(*10*)
Leafy greens					
Sweden	2012–2013	147	0	0	M. Egervärn et al., unpub. data
Imported	2012–2013	375	0.5	0	M. Egervärn et al., unpub. data
Mixed origin	2012–2013	108	0	0	M.Egervärn et al., unpub. data

*ESBL, extended-spectrum β-lactamase; pAmpC, plasmid-encoded AmpC. †These are not samples, but hospital isolates from bloodstream infections submitted to the Public Health Agency of Sweden and confirmed as producers of ESBL and pAmpC. ‡Only samples from chicken meat from Denmark.

Isolates from **broilers and chicken meat in Sweden obtained during 2010, community carriers, and bloodstream infections were subjected to ESBL/pAmpC gene sequencing, multilocus sequence typing (MLST), transfer of plasmids, and subsequent plasmid replicon typing** (*7**–**9*) **(S. Ny et al., unpub. data). When data was lacking in previous studies** (*10**,**14**,**15*; **M. Egervärn et al., unpub. data), analyses were performed in our study as described.**

## Characterization of Isolates

MLST was performed by using an MLST Database (http://mlst.warwick.ac.uk/mlst/dbs/Ecoli), and sequence types (STs) were identified by using BioNumerics versions 7.0 or 7.1 (Applied Maths, Ghent, Belgium). Transfer of plasmids carrying genes encoding ESBL/pAmpC was performed by electroporation to ElectroMax DH10B cells (Life Technologies, Carlsbad, CA, USA), and transformation of plasmids was confirmed by detection of genes as described (*8*). Plasmid replicon typing was performed on transformants by using the PBRT Kit (Diatheva, Fano, Italy). For transformants positive for incompatibility group *inc*I1, the plasmid was subjected to plasmid MLST (pMLST) by using a Plasmid MLST database (http://pubmlst.org/plasmid/).

## Statistical Analysis

Descriptive statistics were used to describe different datasets. Further analysis was undertaken by creating different profiles on the basis of ST type, replicon type, and ESBL/pAmpC gene.

## Results

### Overlap of Genes Encoding ESBL and pAmpC

**Figure 1:**
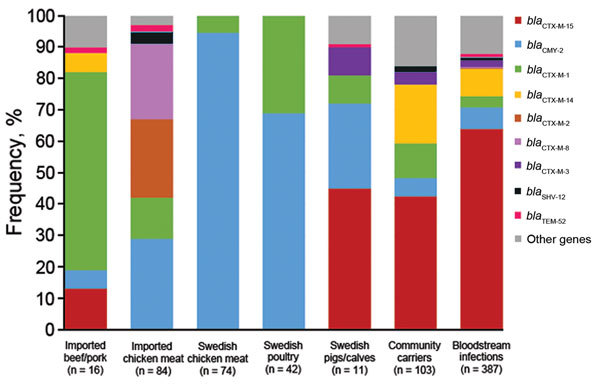
Frequency of overlapping extended-spectrum β-lactamase– and plasmid-encoded AmpC genes in *Escherichia coli* isolates from various sources, Sweden. Data for leafy greens were excluded because there were only 2 isolates (both *bla*_CTX-M-1_) from this source.

Overlap between sectors (humans, farm animals, and foods) was defined as identical genetic traits in at least 1 isolate from human samples and 1 isolate from farm animal or food samples. A total of 24 genes encoding ESBL or pAmpC were identified across all sectors. *bla*_CMY-2_ and *bla*_CTX-M-1_ were the only genes present in all sectors and the only genes identified in poultry from Sweden (laying hens and broilers) and chicken meat (domestically produced) (Figure 1; Technical Appendix). These genes were also commonly detected in chicken meat from Europe. *bla*_CTX-M-1_ was the most common gene in isolates from imported beef/pork, and it was also identified in 2 isolates from imported leafy greens. ESBL/pAmpC-producing isolates in chicken meat from South America had a different gene distribution dominated by *bla*_CTX-M-2_ and *bla*_CTX-M-8_ (Figure 1)_._ *bla*_CMY-2,_ *bla*_CTX-M-1,_ *bla*_CTX-M-2_, and *bla*_CTX-M-8_ were present in isolates from community carriers and bloodstream infections, but were not the most common genes (Figure 1; Technical Appendix).

The 2 most common genes identified in isolates from community carriers and bloodstream infections (*bla*_CTX-M-15_ and *bla*_CTX-M-14_) were not isolated from chicken meat or poultry from Sweden (Figure 1; Technical Appendix). *bla*_CTX-M-15_ was present in isolates from pigs and calves in Sweden (n = 4) and imported beef (n = 2), and *bla*_CTX-M-14_ was detected in 1 isolate from imported pork. Other genes identified in isolates from humans that also were detected in farm animals or meat were *bla*_CTX-M-3_ (pig from Sweden), *bla*_SHV-12_ (imported chicken meat), and *bla*_TEM-52_ (pigs from Sweden, and imported chicken meat and pork) (Figure 1; Technical Appendix).

### Overlap of Plasmid Replicon Types

**Figure 2:**
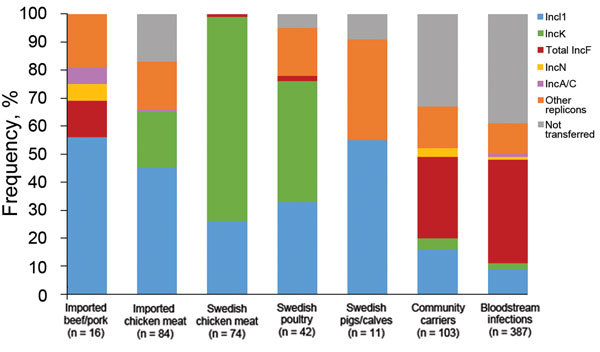
Frequency of overlapping plasmid replicon types containing extended-spectrum β-lactamase– and plasmid-encoded AmpC genes in *Escherichia coli* isolates from various sources, Sweden. Data for leafy greens were excluded because there were only 2 isolates (incI1 and incI2). Other replicon types also include nontypeable plasmids. Gray bar indicates plasmids that could not be transferred by electroporation, primarily isolates with *bla*_CTX-M-15_ and *bla*_CTX-M-2_.

We found a difference between sectors with regards to replicon types of plasmids carrying ESBL/pAmpCs genes (Figure 2). In isolates from chicken meat and poultry from Sweden, incI1 and incK plasmids were primarily detected. IncI1 was the most common replicon type in isolates from imported beef/pork. IncI1 and incK plasmids were also detected in isolates from humans, but to a lesser extent (Figure 2). Isolates from humans had mainly plasmids belonging to different incF replicon types, which were present only occasionally in isolates from poultry in Sweden, chicken meat, and imported beef/pork. Some plasmids could not be transferred by electroporation. This finding was especially common for human isolates carrying *bla*_CTX-M-15_ and isolates carrying *bla*_CTX-M-2_ from imported chicken meat **(S. Ny et al., unpub. data)** (*10*).

### Overlap of Plasmids and Genes Encoding ESBL and pAmpC

The following combinations found in foods or farm animals were also identified in humans isolates: incK-*bla*_CMY-2_, incI1-*bla*_CMY-2_, incI1- *bla*_CTX-M-1_, incI1-*bla*_CTX-M-15_, incI1-*bla*_CTX-M-8_, incFII-*bla*_CTX-M-1,_ incFII-*bla*_CTX-M-14,_ incI1-*bla*_TEM-52_, incN-*bla*_CTX-M-1_, and incA/C-*bla*_CMY-2_ (Technical Appendix). In poultry and chicken meat from Sweden, the combinations incK-*bla*_CMY-2_, incI1-*bla*_CMY-2_, and incI1- *bla*_CTX-M-1_ were primarily identified. These combinations were also identified in imported chicken meat and pigs/calves from Sweden (Technical Appendix). In isolates from humans, these 3 combinations constituted in 9% and 4% of isolates from community carriers and bloodstream infections respectively. The combination incI1-*bla*_CTX-M-8_ was identified in chicken meat from South America and was also identified in 2 isolates from bloodstream infections (Technical Appendix) **(S. Ny et al., unpub. data)**.

The 2 most common combinations identified in isolates from humans that overlapped with farm animals or foods were incI1-*bla*_CTX-M-15_ and incFII-*bla*_CTX-M-15._ These combinations were identified in 1 isolate from pig/calves in Sweden and 2 isolates from imported beef/pork (Technical Appendix).

Because incI1 plasmids with identical genes overlapped in different sectors (Technical Appendix), pMLST was performed to further discriminate between incI1 plasmids. Eight different overlapping incI1 plasmid STs (pSTs) were identified in isolates from community carriers (6 isolates), bloodstream infections (10 isolates), poultry from Sweden (15 isolates), chicken meat from Sweden (17 isolates), imported chicken meat (23 isolates), calves/pigs from Sweden (2 isolates), imported beef/pork (7 isolates), and leafy greens (1 isolate) (Table 2).

**Table 2:** Overlapping plasmid multilocus sequence types for 114 incI1 plasmids carrying ESBL/pAmpC genes in *Escherichia coli* isolates from various sources, Sweden*

Plasmid multilocus sequence type	ESBL/pAmpC gene	Category (no.)	*E. coli* multilocus sequence type
3	*bla*_CTX-M-1_	Community carriers (4), bloodstream infections (1), poultry (9), chicken meat from Sweden (3), imported chicken meat (2), imported beef/pork (4), leafy greens (1)	57, 80, 88, 117, 131, 135, 141,155, 219, 602, 752, 744, 847, 1335 1594,1607,1640, 4162, 4367
3-like†	*bla*_CTX-M-1_	Bloodstream infections (1), poultry (3)	62,155
7	*bla*_CTX-M-1_	Blood stream infections (3), imported chicken meat (5)	117, 453, 746, 752, 2500, 4373
31	*bla*_CTX-M-15_	Community carriers (1), bloodstream infections (1), pigs or calves (1), imported beef/pork (2)	10, 162, 349
114	*bla*_CTX-M-8_	Bloodstream infections (2), imported chicken meat (9)	10, 155, 533 1304, 1773, 4362
36	*bla*_TEM-52_	Bloodstream infections (1), imported chicken meat (2), pigs or calves (1), imported pork/beef (1)	10, 86,155, 297, 2169
2	*bla*_CMY-2_	Bloodstream infections (1), chicken meat from Sweden (2)	10, 58, 299
12	*bla*_CMY-2_	Community carriers (1), poultry (3), chicken meat from Sweden (12), imported chicken meat (5)	10, 38, 48, 58, 69, 117, 131, 206, 297, 1079

*ESBL, extended-spectrum β-lactamase; pAmpC, plasmid-encoded AmpC. †The *sog*S gene could not be detected by PCR.

### Overlap of *E. coli* STs and Plasmid Genes Encoding ESBL and pAmpC

When we compared clonal distributions on the basis of *E. coli* MLST, plasmid replicon type, and ESBL/pAmpC genes, 3 overlapping combinations (ST155-incI1-*bla*_CXT-M-1,_ ST10-incI1-*bla*_CXT-M-15_ and ST57-incK-bla_CMY-2_) were identified (online Technical Appendix). These 3 combinations constituted 17 (2%) of 715 isolates tested. For the combination ST155-incI1-*bla*_CXT-M-1_, incI1 from community carriers and imported chicken meat belonged to pST3, and the 3 incI1 plasmids from poultry in Sweden belonged to a pST3-like incI1 (nontypeable *sog*S gene) (Table 2). The incI1 in the isolate from bloodstream infections with the overlapping combination ST10-incI1-*bla*_CXT-M-15_ belonged to pST172, and the others belonged to pST37 (community carriers) and pST31 (calves from Sweden and imported beef). This finding decreased the total number of overlapping clones from 17 to 11: 2 from community carriers, 3 from imported chicken meat, and 6 from poultry in Sweden. Thus, the only clonal overlap identified consisted of isolates from farm animals, foods and community carriers.

## Discussion

ESBL/pAmpC-producing *E. coli* in food and farm animals in Sweden appears to have had a limited effect on presence among human community carriers and the increasing problem with ESBL/pAmpC in healthcare facilities in this country, as shown in this study by low clonal overlap of isolates from community carriers, imported chicken meat, and poultry from Sweden. In addition, no overlap of isolates from bloodstream infections and isolates from foods or animals was identified. Thus, our findings confirmed results of previous analysis of risk factors associated with community carriage in Sweden, which indicated that preferred diets of humans do not increase the risk of becoming a carrier of ESBL/pAmpC-producing *E. coli* **(S. Ny et al., unpub. data)**. However, one risk factor identified for being a community carrier was travel to Asia and Africa, which is supported by previous studies showing an increased carriage rate in healthy residents of Sweden after visits to these areas (*16**,**17*).

The finding that farm animals are a limited dissemination route to humans has also been reported in a study conducted in the United Kingdom, the Netherlands, and Germany, which showed limited overlap of ESBL-positive *E. coli* isolates of human and animal origin (*18*). However, another study in Germany reported a major set of similar subtypes of ESBL-positive *E. coli* (*11*). Unfortunately it is difficult to make further comparisons between the results of the current study and these 2 studies because different molecular methods were used (*11**,**18*). In addition, the other 2 studies did not include pAmpC-producing *E. coli* in their evaluations.

Nonetheless, results of the current study indicate that limited dissemination of ESBL/pAmpC genes or plasmids could have occurred between sectors. This conclusion is based on the fact that 4 plasmid/gene combinations (incK-*bla*_CMY-2,_ incI1-*bla*_CMY-2_, incI1- *bla*_CTX-M-1_, and incI1-*bla*_CTX-M-8_) identified in isolates from humans can be considered to be animal associated. IncK-*bla*_CMY-2_ and incI1-*bla*_CMY-2_ was the most prevalent combination in isolates from poultry in Sweden and domestically produced chicken meat and were also found in pigs and calves in Sweden.

Furthermore, IncI1-*bla*_CTX-M-1_ and incI1-*bla*_CTX-M-8_ were common in imported foods, primarily chicken meat, and incI1-*bla*_CTX-M-1_ was detected in farm animals and chicken meat in Sweden. It has also been suggested that incI1 and incK plasmids might have emerged from animal reservoirs (*19*). Results of our study support this suggestion because a clear difference in plasmids carrying the genes encoding ESBL/pAmpC from farm animal/foods and human isolates was observed (Figure 2). The hypothesis that ESBL/pAmpC genes and plasmids from foods/animals could have disseminated to humans in Sweden is further supported by the fact that incI1 plasmids with the same pMLST types were identified in different sectors (Table 2; Technical Appendix).

In our study, incI1-pST3-*bla*_CTX-M-1_ and incI1-pST12-*bla*_CMY-2_ plasmids were identified in isolates from humans, farm animals, and foods. IncI1-pST3-*bla*_CTX-M-1_ is of particular interest because it has been commonly identified in poultry in Europe and might have spread to other animal species and humans (*12**,**20**,**21*). Another example of possible ESBL/pAmpC plasmid spread from foods to humans was detection of 2 bloodstream infection isolates carrying incI1-pST114-*bla*_CTX-M-8_ (Table 2). A recent study in the Netherlands that used whole-genome sequencing also concluded that it is not clonal dissemination, but rather plasmids and genes that are being disseminated between human and animals, mainly poultry (*22*).

The results of this study also confirm the conclusion of the EFSA that chicken meat can be a dissemination route for ESBL/pAmpC to humans, with poultry serving as the reservoir (*1*). However, this reservoir so far appears to have had a restricted effect on bloodstream infections and community carriers in Sweden. Only 2 of 103 isolates from community carriers were identified as possibly being associated with poultry on the basis of ST plasmid–gene combinations of isolates. Our results indicate that, in Sweden, 0.09% of the population might be expected to carry poultry-associated isolates. In addition, only 3% of isolates from bloodstream infection and 5% from community carriers carried identical plasmid–gene combinations to those identified in isolates from chicken meat and poultry in Sweden.

These results contradict the findings of studies in the Netherlands, which report larger clonal overlap (>40%) of ESBL-producing isolates (pAmpC was not included) from humans, broilers, and chicken meat (*12**,**13*). Two other studies from the Netherlands reported that 19% of human clinical isolates have identical plasmid-ESBL gene combinations and 4% plasmid-*bla*_CMY-2_ combinations as those identified for poultry (*12**,**23*). The considerable difference between Sweden and the Netherlands is difficult to explain, but it could be influenced by differences in human and animal population densities, farming intensity, climate, biosafety, and hygiene practices. The low rate of antimicrobial drug use in poultry in Sweden compared with that in the Netherlands might have also influenced the differences (*6*). With regards to isolates from pigs/calves and imported pork/beef, these isolates had gene and plasmid–gene combinations comparable with those of human isolates. In addition, prevalences were much lower in isolates from pigs/calves and imported pork/beef than in chicken meat/poultry. Thus, it is likely that most of these isolates could be examples of human-to-animal spread.

The results of our study suggest that, to control the increase in ESBL/pAmpC in the human healthcare system in Sweden, minimizing transmission between humans should be prioritized. However, the high prevalence of ESBL/pAmpC in chicken meat and poultry in Sweden is problematic, and precautionary efforts should be made to reduce this prevalence in Sweden and internationally. Changes in the molecular epidemiology of ESBL/pAmpC might occur quickly, and foods and farm animals could play a more critical role in the near future. Therefore c**ontinuous monitoring and comparative analyses of ESBL/pAmpC from farm animals, foods, and humans, as well as limiting spread within and between different sectors are needed. To reduce the frequency and transfer of ESBL/pAmpC, continued collaboration between professionals and agencies working in human healthcare, animal healthcare, and the food industry is needed. In addition, collaboration with environmental professionals is also essential.**

**In Sweden, foods and farm animals appear to be limited contributors to ESBL/pAmpC-producing *E. coli* in community carriers and its increasing prevalence in human bloodstream infections. However, foods might function as a dissemination route, and farm animals might function as a potential reservoir for genes encoding ESBL/pAmpC and plasmids carrying these genes. On a gene/plasmid level, there is an overlap between food/farm animals, primarily poultry, and humans, but compared with results from other studies in Europe, this overlap is limited.**

**Technical Appendix.** Plasmid and clonal overlap between sectors (humans, farm animals, and foods) in *Escherichia coli* isolates, Sweden.
