# Supplementary material for: Limited Dissemination of Extended-Spectrum β-Lactamase– and Plasmid-Encoded AmpC–Producing Escherichia coli from Food and Farm Animals, Sweden
Source: Emerg Infect Dis. 2016 Apr;22(4):634–40. doi: 10.3201/eid2204.151142 (PMC4806949; doi:10.3201/eid2204.151142)
Supplement: Supplementary file 1 — Technical Appendix. Plasmid and clonal overlap between sectors (humans, farm animals, and foods) in Escherichia coli isolates, Sweden. [file 15-1142-Techapp-s1.pdf]

Article DOI: http://dx.doi.org/10.3201/eid2204.151142

Dissemination of Extended-Spectrum β-Lactamase– and Plasmid-Encoded AmpC–Producing *Escherichia coli* from Food, Sweden

Technical Appendix.

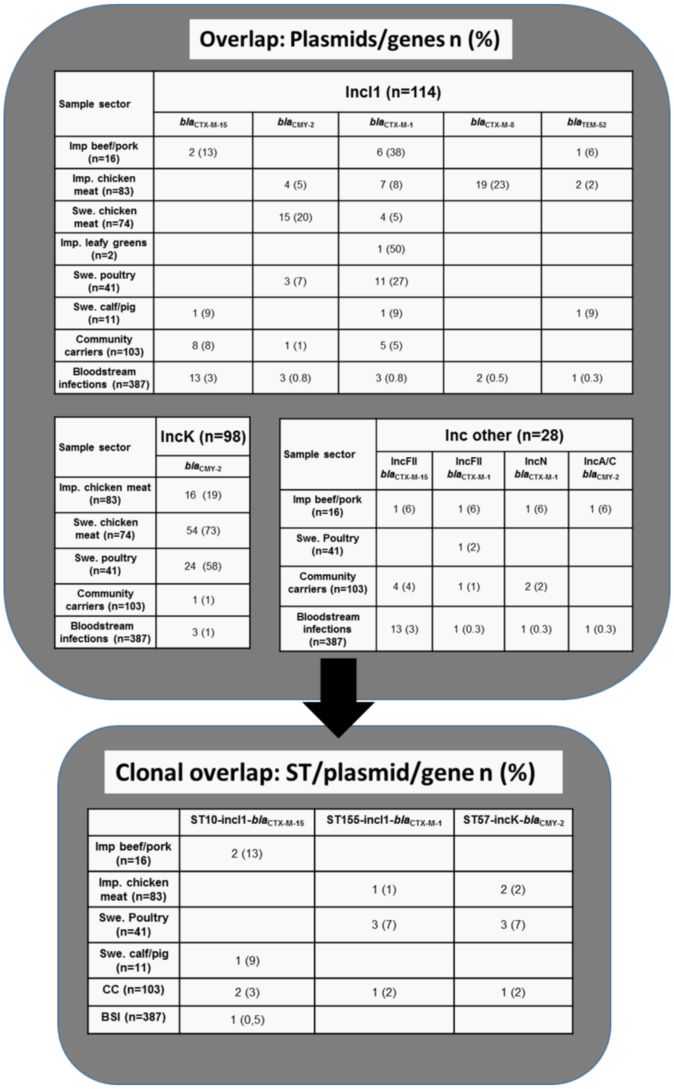

Overlap: Plasmids/genes n (%)

Sample sector	IncI1 (n=114)				
	bla$_{CTX-M-15}$	*bla*$_{CMY-2}$	*bla*$_{CTX-M-1}$	*bla*$_{CTX-M-8}$	*bla*$_{TEM-52}$
Imp beef/pork (n=16)	2 (13)		6 (38)		1 (6)
Imp. chicken meat (n=83)		4 (5)	7 (8)	19 (23)	2 (2)
Swe. chicken meat (n=74)		15 (20)	4 (5)		
Imp. leafy greens (n=2)			1 (50)		
Swe. poultry (n=41)		3 (7)	11 (27)		
Swe. calf/pig (n=11)	1 (9)		1 (9)		1 (9)
Community carriers (n=103)	8 (8)	1 (1)	5 (5)		
Bloodstream infections (n=387)	13 (3)	3 (0.8)	3 (0.8)	2 (0.5)	1 (0.3)

Sample sector	IncK (n=98)
	bla$_{CMY-2}$
Imp. chicken meat (n=83)	16 (19)
Swe. chicken meat (n=74)	54 (73)
Swe. poultry (n=41)	24 (58)
Community carriers (n=103)	1 (1)
Bloodstream infections (n=387)	3 (1)

Sample sector	Inc other (n=28)			
	IncFII *bla*$_{CTX-M-15}$	IncFII *bla*$_{CTX-M-1}$	IncN *bla*$_{CTX-M-1}$	IncA/C *bla*$_{CMY-2}$
Imp beef/pork (n=16)	1 (6)	1 (6)	1 (6)	1 (6)
Swe. Poultry (n=41)		1 (2)		
Community carriers (n=103)	4 (4)	1 (1)	2 (2)	
Bloodstream infections (n=387)	13 (3)	1 (0.3)	1 (0.3)	1 (0.3)

Clonal overlap: ST/plasmid/gene n (%)

	ST10-incI1-*bla*$_{CTX-M-15}$	ST155-incI1-*bla*$_{CTX-M-1}$	ST57-incK-*bla*$_{CMY-2}$
Imp beef/pork (n=16)	2 (13)		
Imp. chicken meat (n=83)		1 (1)	2 (2)
Swe. Poultry (n=41)		3 (7)	3 (7)
Swe. calf/pig (n=11)	1 (9)		
CC (n=103)	2 (3)	1 (2)	1 (2)
BSI (n=387)	1 (0,5)		

Technical Appendix Figure. Plasmid and clonal overlap between sectors (humans, farm animals, and foods) in *Escherichia coli* isolates, Sweden. Plasmid overlap (plasmid replicon type/extended-spectrum β-lactamase [ESBL]/plasmid-encoded AmpC [pAmpC] gene) and clonal overlap (*E. coli* multilocus sequence type/plasmid replicon type/ESBL/pAmpC gene) were defined as identical genetic traits in at least 1 isolate from human samples and 1 isolate from farm animal or food samples. Imp, imported; Swe., Swedish; ST, sequence type; CC, clonal complex; BSI, bloodstream infection.
